# Supplementary material for: Learnings from Separate Aconitum Poisonings in British Columbia and Ontario, Canada in 2022
Source: Toxins (Basel). 2025 Mar 7;17(3):125. doi: 10.3390/toxins17030125 (PMC11946634; doi:10.3390/toxins17030125)
Supplement: Supplementary file 1 [file toxins-17-00125-s001.zip › toxins-3503356-supplementary.pdf]

Learnings from Separate *Aconitum* Poisonings in British Columbia and Ontario, Canada in 2022

Lorraine McIntyre, Stefanie Georgopoulos, Dorianna Simone, Emily Newhouse, JoAnne Fernandes, David A. McVea, Arnold Fok, Ania-Maria McIntyre, Bryn Shurmer, Marie-Claude Gagnon, Michael Chan, Marina Chiaravalloti, Nikita Saha Turna, Debra Kent, Dennis Leong, Katherine Paphitis, Christina Lee and the Outbreak Investigation Teams

Table S1. Roles, acronyms and jurisdiction during aconitine investigations in British Columbia and Ontario

**Investigation Activities**	**Groups involved in outbreak response for the provinces of British Columbia (BC) and Ontario (ON)** (when present the province is indicated in brackets)
Patient contact and treatment	Emergency Room (ER) physicians[1] (BC; ON); Poison Control Centres (PCC): Drug and Poison Information Centre[2] (DPIC, BC); (PCC, ON)
Collection of samples (clinical, food)	ER physicians[1] (BC; ON), Public Health[1,2] (PH) (BC; ON)
Clinical testing	Forensics laboratory[2] (ON)
Food testing	British Columbia Institute of Technology (BCIT, BC) Natural Health Products Laboratory[1] (NHPL), Canadian Food Inspection Agency (CFIA) toxin and genotyping/botany plant laboratories[3] (BC; ON)
Field Investigation	Public Health (PH): Fraser Health Authority[1] (FHA, BC); York Region Public Health[1] (YRPH, ON)
Product supplier investigation	PH[1] (YRPH, ON), CFIA[3] (BC; ON)
Meeting coordination	BC Centre for Disease Control[2] (BCCDC, BC); Public Health Ontario[2] (PHO, ON), Public Health Agency of Canada-Outbreak Management Division[3] (PHAC-OMD, ON)
Communications of PHA to health partners and PHN to the public	PH[1] (BC; ON), Ministry of Health[2] (MOH, ON), CFIA[3] (ON), PHAC-OMD[3] (ON)
Recall communications and product risk assessments	CFIA-Office of Food Safety and Recall[3] (CFIA-OFSR, ON), Health Canada-Bureau of Chemical Safety[3] (HC-BCS, ON)<br>Canadian Network of Public Health Intelligence (CNPHI)<br>INFOSAN-Food and Agriculture Organization (FAO)/World Health Organization (WHO) International Food Safety Authorities Network
Other acronyms	*Kaempferia galanga* powder (KGP)<br>Medical Health Officer (MHO)[1]<br>Public health alerts (PHA) to health partners<br>Public health notices (PHN) to the public

Jurisdictional roles: [1] – Regional; [2] – Provincial; [3] – Federal.

Table S2. Summarized aconitine outbreak descriptions in two Canadian provinces in 2022

Outbreak synopses for BC aconitine investigation, February 2022	Outbreak synopsis for ON aconitine investigation, August to October 2022
On February 4, 2022, 2 cases of foodborne illness were treated in Burnaby, BC following the consumption of a home-prepared chicken dish containing recently purchased powdered sand ginger (*Kaempferia galanga* powder or KGP) which they reported tasted unusual. Both cases presented to the hospital with cardiovascular irregularity, dizziness, and vomiting within 2 hours of consuming the meal. The cases were monitored and released within 12 hours.<br><br>Emergency room (ER) physicians consulted the poison control centre for advice on diagnosis of the suspect toxin based on symptom presentation. Poison control notified the regional health authority, who conducted a follow-up inspection at the retail store where the product was reported to have been purchased. Inspectors held the remaining product pending the outcome of testing and investigation. Delays in contacting cases and identifying the purchase location hindered the timeliness of on-site inspection.<br><br>Initial testing of KGP for aconitine occurred in a research laboratory. Toxic aconitine alkaloids were detected in the opened package, leading to release of a public health notice (PHN) to the public five weeks after the illnesses occurred. The remaining opened and unopened packages of KGP were sent to federal laboratories and tested for *Aconitum* plant DNA and for aconitine-following method development. Plant DNA testing detected *Aconitum,* and other plant species, however, *Kaempferia galanga* was not detected in the spice packets. High levels of aconitine toxin were detected in one unopened KGP sample, and lower levels were detected in 10 other unopened samples.<br><br>Product handling within the store was investigated. Inspectors discovered that the retailer had prepared 70 g packages labelled as KGP from a 1 lb wholesale bag. Although handling of spice in the shop appeared satisfactory for repackaging, the possibility of in-store contamination of the spice could not be ruled out.	On August 28, 2022, public health received a report of several individuals presenting with severe illness following the consumption of a chicken dish containing KGP at a restaurant. There were 15 individuals and family members in multiple ERs. Investigations identified 11 cases who presented to local hospitals within 5 hours of consuming the restaurant meal, four were admitted to hospital and received intensive care.<br><br>The restaurant was closed while the investigation was ongoing. In collaboration with the restaurant staff, inspectors identified a newly purchased bag of KGP as the plausible ingredient linked to illnesses. KGP was detained and sent for testing. Inspectors continued their investigation at the restaurant and with the product distributor.<br><br>Aconitine was confirmed in the seized KGP and present in blood samples collected from cases. A health risk assessment (HRA) found levels of aconitine in the powder was potentially 600 times greater than what would be considered to have a toxic effect. Import searches for KGP suppliers by federal investigators later found five importers in BC.<br><br>Rapid communications between all local, provincial and federal partners, and the possible implications for jurisdictions outside of ON, led to formation of a national Outbreak Investigation Coordination Committee (OICC) for information sharing among partners. Communications included several public health advisories (PHA) to professionals, PHN, food recall warnings and translations for targeted public populations.<br><br>On October 24, 2022 an additional case of aconite poisoning requiring hospitalization occurred in ON. The KGP was consumed in a home-prepared meal and was purchased before the recall. It shared the same lot code that caused earlier poisonings. The case could not recall where the product was purchased. In response,

There were no other bags of products left remaining on the premises, and there were no in-store records on how many products were sold. The product was received from a distributor located in ON who imported KGP from China.	product recall verification activities and public health warnings were repeated. No products were found available for sale.
